# Cross Neutralization of Afro-Asian Cobra and Asian Krait Venoms by a Thai Polyvalent Snake Antivenom (Neuro Polyvalent Snake Antivenom)

**DOI:** 10.1371/journal.pntd.0001672

**Published:** 2012-06-05

**Authors:** Poh Kuan Leong, Si Mui Sim, Shin Yee Fung, Khomvilai Sumana, Visith Sitprija, Nget Hong Tan

**Affiliations:** 1 Department of Pharmacology, Faculty of Medicine, University of Malaya, Kuala Lumpur, Malaysia; 2 CENAR and Department of Molecular Medicine, Faculty of Medicine, University of Malaya, Kuala Lumpur, Malaysia; 3 Queen Saovabha Memorial Institute, Bangkok, Thailand; University of Kelaniya, Sri Lanka

## Abstract

**Background:**

Snake envenomation is a serious public health threat in the rural areas of Asian and African countries. To date, the only proven treatment for snake envenomation is antivenom therapy. Cross-neutralization of heterologous venoms by antivenom raised against venoms of closely related species has been reported. The present study examined the cross neutralizing potential of a newly developed polyvalent antivenom, termed Neuro Polyvalent Snake Antivenom (NPAV). NPAV was produced by immunization against 4 Thai elapid venoms.

**Principal Findings:**

*In vitro* neutralization study using mice showed that NPAV was able to neutralize effectively the lethality of venoms of most common Asiatic cobras (*Naja* spp.), *Ophiophagus hannah* and kraits (*Bungarus* spp.) from Southeast Asia, but only moderately to weakly effective against venoms of *Naja* from India subcontinent and Africa. Studies with several venoms showed that the *in vivo* neutralization potency of the NPAV was comparable to the *in vitro* neutralization potency. NPAV could also fully protect against *N. sputatrix* venom-induced cardio-respiratory depressant and neuromuscular blocking effects in anesthetized rats, demonstrating that the NPAV could neutralize most of the major lethal toxins in the *Naja* venom.

**Conclusions/Significance:**

The newly developed polyvalent antivenom NPAV may find potential application in the treatment of elapid bites in Southeast Asia, especially Malaysia, a neighboring nation of Thailand. Nevertheless, the applicability of NPAV in the treatment of cobra and krait envenomations in Southeast Asian victims needs to be confirmed by clinical trials. The cross-neutralization results may contribute to the design of broad-spectrum polyvalent antivenom.

## Author Summary

Snake envenomation is a serious public health threat in the rural areas of Asia and Africa. To date, the only proven treatment for snake envenomation is antivenom therapy. Owing to the difficulties in the diagnosis of the biting species, there is a need to develop polyvalent antivenoms that could cross-neutralize venoms of medically important venomous snakes in the various regions. Recently, Thai Red Cross Society from Thailand has developed a new polyvalent antivenom for treatment of cobra and krait venoms. The polyvalent antivenom, termed “Neuro Polyvalent Snake Antivenom (NPAV),” is raised against venoms of two Thai cobras and two Thai kraits. Our results indicated that the polyvalent antivenom can effectively neutralize venoms from many Southeast Asian cobras, kraits and king cobra but is less effective against Indian cobra venoms. Studies using anesthetized rats showed that NPAV can effectively protect against cobra venom-induced cardio-respiratory depressant and neuromuscular blocking effects, confirming that the antivenom can effectively neutralize the major lethal toxins of common cobra venoms. This new antivenom may find potential application in the treatment of elapid bites in Southeast Asia, especially Malaysia, a neighboring nation of Thailand.

## Introduction

The global figure of snake envenoming cases has been estimated to be greater than 1.8 million annually, with an annual death toll of more than 90,000. Most of the snake envenoming cases occurs in South Asia and Southeast Asia (estimated 720,000 cases, 53,000 fatality), followed by Africa (estimated 420,000 cases, 32,000 fatality) [1], and the main biting species are snakes from the Elapidae and Viperidae families. Among members of the Elapidae family, the cobras and kraits are the main causes of snake envenoming [2], [3]. There are about 34 species of cobras belonging to 7 genera (*Aspidelaps*, *Boulengerina*, *Hemachatus*, *Naja*, *Ophiophagus*, *Pseudohaje* and *Walterinnesia*). The genus *Naja* distributed extensively across large regions of the Africa (13 species) and Asia (12 species) [4]. *Ophiophagus hannah* or commonly known as the king cobra, is the only member of the *Ophiophagus* genus and is found only in Asia. *Bungarus* (the kraits), are represented by 12 species and their distribution is confined to the Indian subcontinent, Southeast Asia, as well as Southern China and Taiwan [4]. Cobra and krait envenomations are generally characterized by neurotoxic envenoming [5].

Antivenom therapy is the only effective treatment for snake envenomation. Monovalent antivenoms are raised with venom from one particular species and hence generally only effective in the treatment of envenomation caused by the particular species. Because of the difficulties in accurate diagnosis of the biting species, polyvalent antivenoms that offer paraspecific protection against several venomous snake bites have also been developed and become commercially available. It has been argued that monovalent antivenoms are generally more effective than polyvalent antivenoms, though this has not been firmly established. At present, several types of polyvalent antivenoms against Afro-Asian venomous snakes are available in the market, produced mainly by Asian or African commercial pharmaceutical firms or government institutions [5], [6]. There is, however, a lack of rigorous evaluation of the paraspecific protective actions of these commercially available polyvalent antivenoms. Recently, Thai Red Cross Society produced a new polyvalent antivenom that offers protection against neurotoxic envenomations by elapids in Thailand. This polyvalent antivenom, termed Neuro Polyvalent Snake Antivenom (abbreviated as NPAV) is raised against venoms of four medically important cobras and kraits in Thailand, i.e. *Naja kaouthia* (Thai monocellate cobra), *Ophiophagus hannah* (king cobra), *Bungarus candidus* (Malayan krait) and *Bungarus fasciatus* (banded krait). In this paper, we report evaluation of the cross-neutralizing potential of NPAV against heterologous venoms of common Afro-Asian cobras (*Naja* spp. and *Ophiophagus hannah*) and Asian kraits (*Bungarus* spp.) We also compared the efficacy of the polyvalent antivenom versus the relevant monovalent antivenom. The results will provide preliminary information as to whether the polyvalent antivenom could find therapeutic application for cobra and krait envenomations outside of Thailand, as well as contribute to the design of a broad-spectrum, Pan-Asian polyvalent antivenom [7].

## Materials and Methods

### Venoms and antivenoms

Venoms of *Naja sputatrix*, *Naja siamensis*, *Naja kaouthia* (Thailand), *Naja philippinensis*, *Naja oxiana*, *Naja atra*, *Naja naja* (Sri Lanka, sample 1), *Naja naja* (India, sample 1 and 2), *Naja melanoneuca*, *Naja nigricollis*, *Naja nubiae*, *Naja katiensis*, *Naja haje*, *Bungarus multicinctus* and *Bungarus caeruleus* were purchased from Latoxan (Valence, France). Venoms of *Naja sumatrana*, *Naja kaouthia* (Malaysia), *Ophiophagus hannah*, *Bungarus candidus*, *Bungarus fasciatus* and *Bungarus flaviceps* were pooled samples obtained from several adult individuals captured in Malaysia whilst *Naja naja* (Sri Lanka sample 2) was a pooled sample obtained from several adult individuals captured in Sri Lanka. After extraction, the venoms were instantly lyophilized. Two antivenoms were studied: (a) Neuro Polyvalent Snake Antivenom (NPAV) (Lyophilised; Batch no. 0030208; Exp. Date April 21st, 2013), a purified F(ab′)_2_ obtained from serum of equines hyperimmunized against a mixture of four venoms: *Naja kaouthia* (Thai monocellate cobra), *Ophiophagus hannah* (king cobra), *Bungarus candidus* (Malayan krait) and *Bungarus fasciatus* (banded krait); (b) *Naja kaouthia* monovalent antivenom (NKMAV) (Full name: Cobra antivenin; Lyophilised; Batch no. 0090406; Exp. Date August 31st, 2014), a purified F(ab′)_2_ obtained from serum of equines hyperimmunized specifically against the venom of Thai *N. kaouthia*. Both of these antivenoms are produced by Queen Saovabha Memorial Institute (QSMI), the Thai Red Cross Society from Bangkok, Thailand. For neutralization studies, both antivenoms were reconstituted in the same manner: 10 mL of normal saline was added to 1 vial of the freeze-dried antivenom. According to the attached fact sheet, 1 mL of the NPAV antivenom is able to neutralize the following amount of snake venoms: 0.6 mg each of *N. kaouthia* and *B. fasciatus* venoms, 0.4 mg of *B. candidus* and 0.8 mg of *O. hannah* venoms; while 1 mL of the NKMAV can neutralize 0.6 mg of *N. kaouthia* venom.

### Animals

Albino mice (ICR strain, 20–25 g) and male Sprague Dawley rats (250–300 g) were supplied by the Laboratory Animal Centre, Faculty of Medicine, University of Malaya. The animals were handled according to the guidelines given by CIOMS on animal experimentation [8]. All experiments involving animals were approved by the Animal Care and Use Committee (ACUC) of the University of Malaya (Ethical clearance letter No. PM/03/03/2010/FSY(R)).

### Determination of protein content

Protein content was determined by Bradford method [9]. All measurements were performed in triplicate. Bovine serum albumin (Sigma, USA) was use to generate a standard curve.

### Chromatographic and electrophoretic profiling of the antivenom

SDS-polyacrylamide gel electrophoresis (SDS-PAGE) was conducted according to the method of Studier [10], using the Bio-Rad broad-range prestained SDS-PAGE standards (6.5–200 kDa) and 15 µL of each antivenom sample (3 mg/mL) was loaded in the gel (12.5%). High performance gel filtration chromatography of the reconstituted antivenom (100 µL, 10 mg/mL) was performed using a Superdex 200 HR 10/30, 13 µm SEC 10×300 mm (GE Healthcare, Sweden). Elution buffer was 100 mM sodium phosphate, 0.15 M NaCl, pH 7.4 at a flow rate of 0.75 mL/min. Protein was monitored by absorbance measurement at 280 nm. The column was calibrated using the following protein standards obtained from Bio-Rad (BIO-RAD Gel filtration Standard): thyroglobulin (670 kDa), γ-globulin (158 kDa), ovalbumin (44 kDa) and myoglobin (17 kDa).

### Determination of venom lethality

The median lethal dose, LD_50_, of the venom was determined by intravenous or intramuscular injection into ICR mice (20–25 g, n = 4). The survival ratio was recorded after 48 h to determine the LD_50_.

### *In vitro* neutralization of lethality of venoms

*In vitro* neutralization of lethality was conducted as described by Ramos-Cerrillo et al. [11]. Briefly, a challenge dose of the venom in 50 µL saline was pre-incubated at 37°C for 30 min with various dilutions of the reconstituted antivenom (NPAV or NKMAV) in normal saline, to give a total volume of 250 µL. The mixture was subsequently centrifuged at 10000× g before being injected into the caudal vein of the mice. The number of survival after 48 h was recorded. Generally, the challenge dose used was 5 LD_50_. However, if 200 µL of the reconstituted antivenom (maximum permitted volume to inject into the mouse) failed to give full protection of the mice, a lower challenge dose of 2.5 LD_50_ was used instead. The antivenom was considered ineffective when none of the animals injected with the pre-incubated mixture (containing 2.5 LD_50_ challenge venom in 50 µL saline and 200 µL of the undiluted reconstituted antivenom) survived. Neutralizing potency of the antivenom was expressed as ED_50_ (the amount of reconstituted antivenom in µL or the ratio of mg venom/mL reconstituted antivenom that gives 50% survival of the animals tested) as well as in term of ‘neutralization potency’ (P, the amount of venom that is completely neutralized by a unit volume of antivenom) calculated according to Morais et al. [12].

### *In vivo* neutralization of lethality of venom

This was carried out by intramuscular injection of 5 LD_50_ or 2.5 LD_50_ of the venom into mice (n = 4) followed by intravenous injection of 200 µL of appropriately diluted reconstituted antivenom, 10 min later. The number of survival after 48 h was recorded.

### Protective actions of the antivenom against *N. sputatrix* venom-induced cardiovascular depressant and neuromuscular blocking effects in anesthetized rats

The study was conducted on three groups of rats (n = 3, 250–300 g) anesthetized with intraperitoneal injection of urethane (1.4 g/kg, *i.p.*) to the point of loss of the eyelid reflex and the pedal withdrawal reflex on painful stimuli. The anesthetized animals were surgically prepared for the simultaneous measurement of blood pressure, heart rate, respiratory rate and muscle twitch tension. Data collection and analysis were conducted using PowerLab 4/30 data Acquisition system equipped with LabChart software (AD Instruments, Australia). Rats in group 1 (termed ‘saline/-’ group) were injected with 50 µL saline intramuscularly at 0 min and served as control; Rats in group 2 (termed ‘NsV/-’ group) were injected with 6 mg/kg *N. sputatrix* venom (dissolved in 50 µL saline) intramuscularly; and rats in group 3 (termed ‘NsV/NPAV’ group) were injected with the same dose of venom intramuscularly followed by intravenous administration of 3 mL of the reconstituted NPAV (1 mL each at 10 min, 30 min and 50 min post-injection of the venom). The volume of antivenom administered and the time points were chosen to ensure no disturbance of the blood pressure, heart rate and respiratory rate occurred.

### Statistical analysis

LD_50_ of the venoms and ED_50_ of antivenoms are expressed as means with 95% confidence intervals (C.I.). LD_50_, ED_50_ (median effective dose) and the 95% confidence intervals (C.I.) were calculated using the probit analysis method of Finney [13] with the BioStat 2009 analysis software (AnalystSoft Inc.). The statistical analysis for pharmacological study was conducted using SPSS. The data (expressed as mean ± S.D.) were analyzed using one-way ANOVA, with Tukey's post hoc multiple-comparison test, with P<0.05 as significant.

## Results

### Composition of the antivenoms

**Figure 1:**
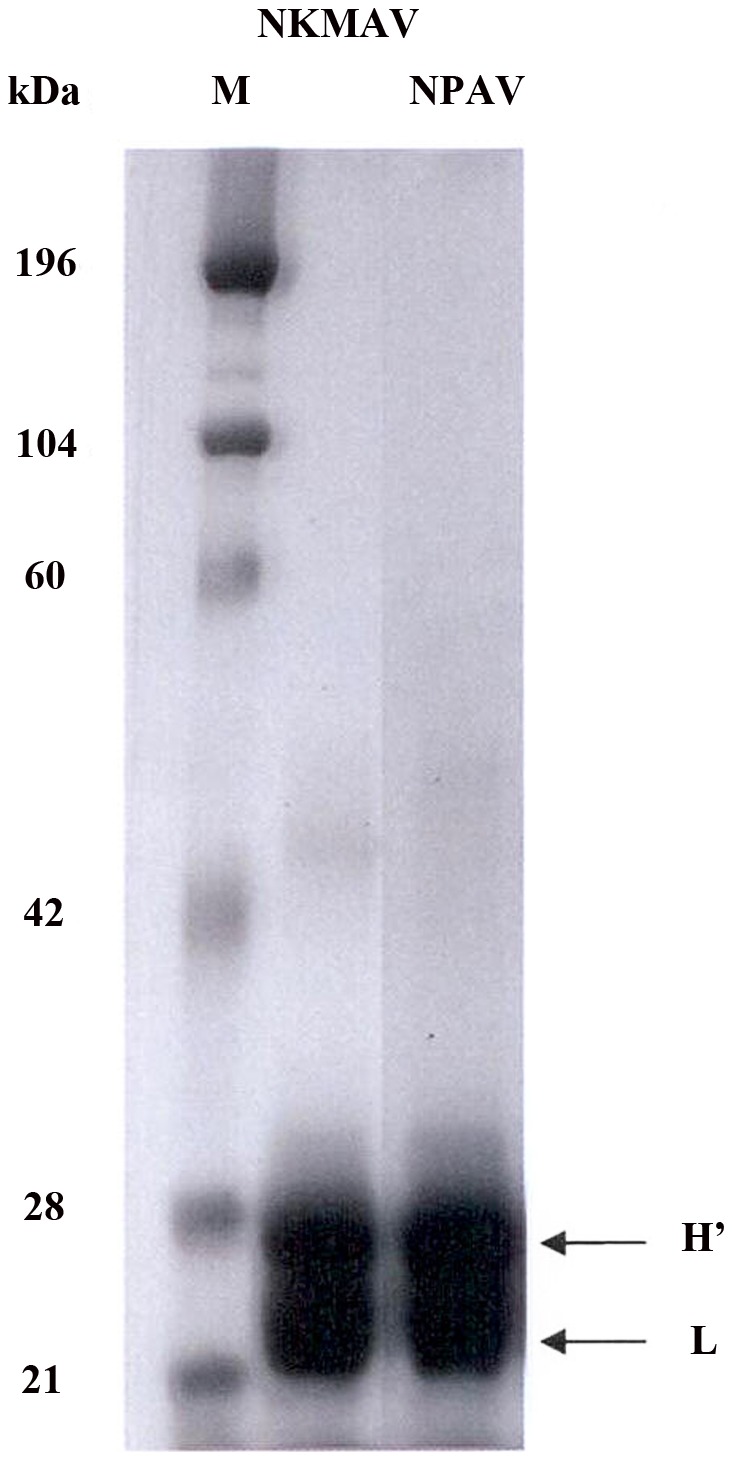
SDS-PAGE of Neuro Polyvalent Snake Antivenom (NPAV) and *Naja kaouthia* monovalent antivenom (NKMAV). Fifteen µL of the respective reconstituted antivenom (3 mg/ml) was loaded on the middle (NPAV) and right lane (NKMAV). Prestained molecular weight markers mixture was loaded on the left lane (M, molecular weight in kDa). The digested heavy chain (H′) and light chains (L) are indicated by black arrowheads.

**Figure 2:**
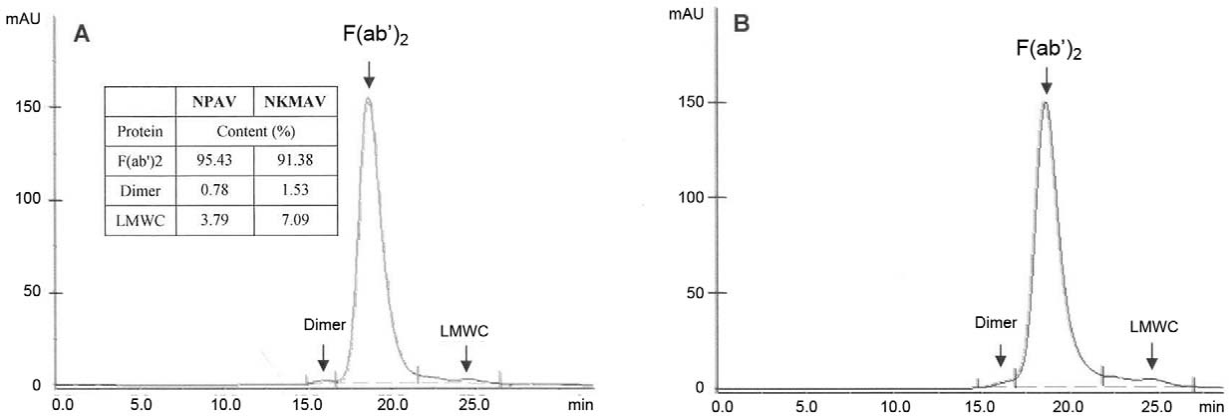
Elution profiles of (A) Neuro Polyvalent Snake Antivenom (NPAV) and (B) *Naja kaouthia* monovalent antivenom (NKMAV). One mg of each sample was applied to Superdex 200 10/300 GL gel column. The arrows identify the peaks. The results of integration of the areas-under-the-curve are shown in the insert table on the upper left. LMWC stands for low molecular weight components.

The protein contents of reconstituted NPAV and NKMAV were 20.3 mg/mL and 12.5 mg/mL, respectively. The SDS-PAGE patterns of NPAV (Neuro Polyvalent Snake Antivenom) and NKMAV (*Naja kaouthia* monovalent antivenom) indicated that there was no distinct band of high or low molecular weight proteins (Fig. 1). The same was also observed in the gel-filtration chromatographic profiles of the two antivenoms (Fig. 2). Based on ‘area-under-the-curve’ comparison, the quantity of F(ab′)_2_ in both these antivenoms were comparable (91–96%) whilst the quantity of respective dimers and low molecular weight proteins in NKMAV were slightly higher than those in NPAV (Figure 2). No high molecular weight aggregates were detected in both of these antivenoms.

### *In vitro* neutralization of venom lethal effects by the antivenoms

The results of the *in vitro* neutralization of venom lethality by the NPAV and the monovalent *N. kaouthia* antivenom (only for selected venoms) are shown in Table 1 and 2, respectively. The results showed that NPAV was able to confer protection/cross-protection against the venoms of all krait as well as almost all the Afro-Asian cobra venoms examined (except for the African spitting cobra *N. katiensis*), although the neutralizing potency range varied from low to high. The NKMAV was able to neutralize the venoms of six Asian cobras (*N. kaouthia* [Thailand & Malaysia], *N. sputatrix*, *N. sumatrana*, *N. siamensis* and *O. hannah*) tested, with efficacy comparable to that of NPAV, but failed to neutralize the venoms of *B. fasciatus* and *B. candidus*. It is interesting to note that our results on the neutralization potentials of NPAV against *N. kaouthia*, *B. fasciatus*, *B. candidus* and *O. hannah* venoms are much higher than stated in the antivenom fact sheet provided, in particular against *O. hannah* venom. It should be noted, however, that the definition of neutralization potential used in the fact sheet has not been clearly stated.

**Table 1:** *In vitro* neutralization of lethality of Afro-Asian cobra and krait venoms by NPAV.

Venom	*i.v.* LD_50_ (µg/g)	Challenge dose	NPAV		
			ED_50_ (µL)	ED_50_ in mg/mL	P (mg/mL)
***Naja sputatrix***	0.90 (0.59–1.36)	5 LD_50_	111.25	0.93 (0.61–1.41)	0.74
***Naja siamensis***	0.28 (0.18–0.42)	5 LD_50_	22.47	1.43 (0.94–2.18)	1.15
***Naja sumatrana***	0.50 (0.40–0.62)	5 LD_50_	25.00	2.30 (1.86–2.85)	1.84
***Naja kaouthia*** ** (Thailand)**	0.23 (0.15–0.34)	5 LD_50_	22.47	1.18 (0.78–1.79)	0.94
***Naja kaouthia*** ** (Malaysia)**	0.89 (0.59–1.35)	5 LD_50_	150.00	0.68 (0.62–0.75)	0.55
***Naja phillipinensis***	0.18 (0.12–0.27)	5 LD_50_	156.57	0.13 (0.11–0.16)	0.10
***Naja atra***	0.56 (0.37–0.84)	2.5 LD_50_	56.00	0.86 (0.79–0.94)	0.52
***Naja oxiana***	1.11 (0.73–1.69)	2.5 LD_50_	37.50	1.70 (1.56–1.85)	1.01
***Naja naja*** ** (India 1)**	1.80 (1.18–2.73)	2.5 LD_50_	200.00	*0.52	0.31
***Naja naja*** ** (India 2)**	1.08 (0.71–1.64)	2.5 LD_50_	156.57	0.40 (0.32–0.49)	0.24
***Naja naja*** ** (Sri Lanka 1)**	1.13 (0.54–2.38)	2.5 LD_50_	100.00	0.65 (0.52–0.84)	0.39
***Naja naja*** ** (Sri Lanka 2)**	1.08 (0.71–1.64)	5.0 LD_50_	89.88	1.39 (0.91–2.08)	1.11
***Naja haje***	0.09 (0.05–1.40)	5.0 LD_50_	78.29	0.13 (0.11–0.16)	0.10
***Naja melanoleuca***	0.33 (0.22–0.51)	5.0 LD_50_	55.63	0.68 (0.44–1.03)	0.54
***Naja nigricollis***	0.75 (0.69–0.82)	2.5 LD_50_	55.63	0.78 (0.49–1.18)	0.47
***Naja nubiae***	0.28 (0.22–0.37)	5.0 LD_50_	78.29	0.41 (0.34–0.50)	0.33
***Naja katiensis***	1.20 (0.97–1.45)	2.5 LD_50_	NE	NE	NE
***Ophiophagus hannah***	1.00 (0.81–1.24)	5.0 LD_50_	11.24	10.23 (6.74–15.54)	8.19
***Bungarus fasciatus***	1.67 (1.10–2.53)	5.0 LD_50_	111.25	1.73 (1.14–2.62)	1.38
***Bungarus candidus***	0.11 (0.07–0.17)	5.0 LD_50_	13.91	0.91 (0.60–1.38)	0.73
***Bungarus flaviceps***	0.175 (0.09–0.21)	5.0 LD_50_	11.24	1.84 (1.21–2.80)	1.47
***Bungarus multicintus***	0.11 (0.05–0.22)	5.0 LD_50_	37.50	0.34(0.31–0.37)	0.27
***Bungarus caeruleus***	0.17 (0.11–0.25)	5.0 LD_50_	78.29	0.12(0.10–0.15)	0.07

For LD_50_ and ED_50_, values in brackets are 95% CI.

2.5 or 5.0 LD_50_ of venom in 50 µL was premixed with 200 µL of NPAV (Neuro Polyvalent Snake Antivenom) and incubated for 30 min. The mixture was then injected into mice (n = 4, 20–25 g) and monitored for 48 h. NE: Not effective at maximum volume of antivenom (200 µL) permitted.

*: : estimated value, as the maximum volume of antivenom (200 µL) only achieved 50% protection of the animals.

**Table 2:** *In vitro* and *in vivo* neutralization of cobra and krait venoms by NPAV and NKMAV.

Venom	*i.v.* LD_50_ (µg/g)	NPAV			NKMAV		
		ED_50_ (µL)	ED_50_ (mg/mL)	P (mg/mL)	ED_50_ (µL)	ED_50_ (mg/mL)	P (mg/mL)
***Naja sputatrix***	0.90 (0.59–1.36)	111.25	0.93 (0.61–1.41)	0.74	111.25	0.93 (0.61–1.41)	0.74
***Naja siamensis***	0.28 (0.18–0.42)	22.47	1.43 (0.94–2.18)	1.15	22.47	1.43 (0.94–2.18)	1.15
***Naja sumatrana***	0.50 (0.40–0.62)	25.00	2.30 (1.86–2.85)	1.84	50.00	1.15 (0.93–1.43)	0.92
***Naja kaouthia*** ** (Thailand)**	0.23 (0.15–0.34)	22.47	1.18 (0.78–1.79)	0.94	22.47	1.18 (0.78–1.79)	0.94
***Naja kaouthia*** ** (Malaysia)**	0.89 (0.59–1.35)	150.00	0.68 (0.62–0.75)	0.55	150.00	0.68 (0.62–0.75)	0.55
***Ophiophagus hannah***	1.0 (0.81–1.24)	11.24	10.23 (6.74–15.54)	8.19	44.94	3.07 (2.80–3.35)	2.46

Abbreviations: NPAV: Neuro Polyvalent Antivenom; NKMAV: *Naja kaouthia* monovalent antivenom.

Data for NPAV are extracted from Table 1 for comparison purpose. For LD_50_ and ED_50_, values in brackets are 95% CI.

Challenge dose for all venoms: 5 LD_50_.

### *In vivo* neutralization of lethal effects of some Asian cobra and krait venoms

Table 3 shows the *in vivo* neutralization of lethality of four venoms from Asian cobras and krait (*N. sputatrix*, *N. kaouthia* (Thailand), *N.kaouthia* (Malaysia) and *B. candidus*) by NPAV. It is interesting to note that the *i.m.*LD_50_ values determined were comparable to the corresponding *i.v.*LD_50_ values. Our results showed that NPAV effectively neutralized all four venoms *in vivo*, with ED_50_ comparable to the corresponding ED_50_ in the *in vitro* neutralization assays.

**Table 3:** *In vivo* neutralization of lethality of some cobra and krait venoms by NPAV.

Species	*i.m.*LD_50_ (µg/g of mouse)	*i.v.*LD_50_ (µg/g of mouse)	*In vivo* Neutralization (ED_50_ in mg/mL venom)	*In vitro* Neutralization (ED_50_ in mg/mL venom)
***Naja sputatrix***	1.41 (1.08–1.85)	0.90 (0.59–1.36)	1.45 (0.96–2.22)	0.93 (0.61–1.41)
***Naja kaouthia*** ** (Thailand)**	0.13 (0.09–0.20)	0.23 (0.15–0.34)	1.59 (1.45–1.89)	1.18 (0.78–1.79)
***Naja kaouthia*** ** (Malaysia)**	1.21 (0.80–1.84)	0.89 (0.59–1.35)	0.88 (0.72–1.09)	0.68 (0.62–0.75)
***Bungarus candidus***	0.17 (0.11–0.25)	0.11 (0.07–0.17)	0.43 (0.29–0.66)	0.91 (0.60–1.38)

In the *in vitro* neutralization experiments, 5 LD_50_ of venom in 50 µL of saline was preincubated with 200 µL of NPAV for 30 min and then injected intravenously into mice (n = 4, 20–25 g). In the *in vivo* neutralization experiments, 5 LD_50_ of venom in 50 µL of saline was injected intramuscularly into mice (n = 4, 20–25 g), and 200 µL of NPAV was injected to the same mouse intravenously, 10 min later. The animals were monitored for 48 h. For LD_50_ and ED_50_, values in brackets are 95% CI.

### The protective effects of NPAV on the *N. sputatrix* venom-induced cardio-respiratory depressant and neuromuscular blocking effects in the anesthetized rats

**Figure 3:**
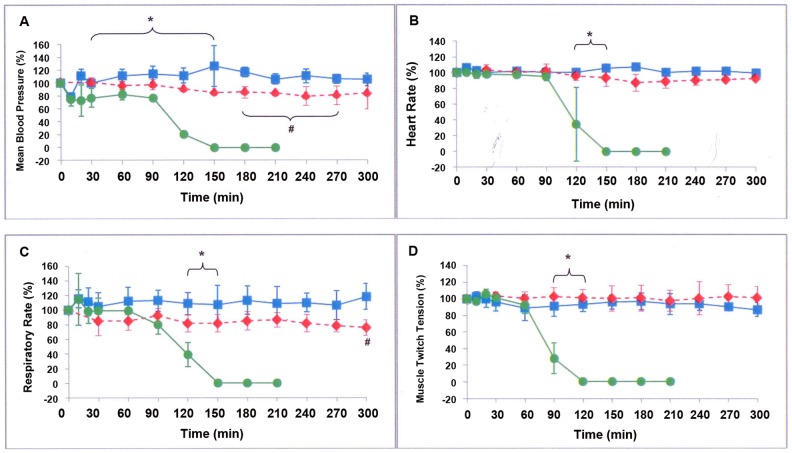
Effects of Neuro Polyvalent Snake Antivenom (NPAV) on the cardio-respiratory depressant and neuromuscular blocking effects. Changes in percentage (%) of (A) mean blood pressure, (B) heart rate, (C) respiratory rate and (D) muscle twitch tension, upon intramuscular injection of *N. sputatrix* venom (6 mg/kg) at 0 min in ‘NsV/-’ group (n = 3; green circle) and ‘NsV/NPAV’ group (n = 3; blue square ); and intramuscular injection of saline in ‘saline/-’ group (n = 3; red diamond) , which served as the control group. The ‘NsV/NPAV’ group received intravenous injection of 1 mL NPAV each at 10, 30 and 50 min (3 mL in total) post venom injection. *P<0.05 by one-way ANOVA between the ‘NsV/-’ group and ‘NsV/NPAV’ group, within the range indicated by the curly bracket. #P<0.05 by one-way ANOVA between the ‘saline/-’ group and ‘NsV/NPAV’ group, within the range indicated by the curly bracket. The animals in ‘NsV/-’ group died within 125–130 min upon venom injection. All values are expressed as mean ± S.D.

Figure 3 shows that NPAV was able to fully protect against the *N. sputatrix* venom-induced cardio-respiratory depressant and neuromuscular blocking effects in the anesthetized rats. The mean blood pressure (BP), heart rate, respiratory rate and muscle twitch tension of the control anesthetized rats (‘saline/-’ group) remained constant for at least 6 hours after the initial stabilization. Following an intramuscular injection of the venom at 4×LD_50_ dose (6 mg/kg), however, there was an immediate small decrease (about 20%) in the BP, which remained constant thereafter for the next 90 min (Figure 3A). During this period, the heart rate remained essentially unaffected (Figure 3B). And then the BP and heart rate both began to fall precipitously from 90 min onward. On the other hand, the respiratory rate and muscle twitch tension were stable only for the first 60 min after venom administration. Both these parameters, however, began to decrease sharply thereafter (Figure 3C and 3D) and death occurred at 125–130 min after the venom injection.

In a parallel series of experiment conducted to examine the ability of NPAV to protect against the cardiovascular depressant and neuromuscular blocking effects of *N. sputatrix* venom, 1 mL each of NPAV was administered via the left jugular vein, at 10, 30 and 50 min, respectively, following the *i.m.* injection of the venom. The results showed that the administration of the antivenom effectively reversed the cardio-respiratory depressant and neuromuscular blocking effects induced by the *N. sputatrix* venom. The heart rate and muscle twitch tension of the antivenom-treated animals (‘NsV/NPAV’ group) were restored to the same levels as the ‘saline/-’ control group (P>0.05, not significantly different between ‘saline/-’ group and ‘NsV/NPAV’ group by one-way ANOVA), whereas the blood pressure and respiratory rate were restored to 80% of the control group (P<0.05 between the two groups) and remained at that level throughout the monitoring period.

## Discussion

### Expression of neutralization capabilities of antivenom

In this report, the neutralization capabilities of the antivenoms are expressed in three different ways: the commonly used median effective dose ED_50_ in µL antivenom, ED_50_ in mg/mL and potency, P, as defined by Morais et al. [12]. For ED_50_, expressing the value in term of mg venom neutralized per mL antivenom is a more realistic assessment of the neutralization capabilities of the antivenom than in term µL antivenom or number of mouse LD_50_, because the LD_50_'s of the various cobra venoms differ substantially. For example, expressed in term of µL antivenom, the ED_50's_ of NPAV against *N. kaouthia* (Malaysia) venom and *N. philippinensis* venom are comparable, but when expressed in term of mg/mL, NPAV was obviously much more effective against *N. kaouthia* (Malaysia) venom then against *N. philippinensis* venom.

Because of the high lethality of certain venoms, a 2.5 LD_50_ instead of the standard 5 LD_50_ was used as the challenge dose. Since the ED_50_ value of an antivenom is highly dependent on the challenge dose, ED_50_ obtained from the two different challenge doses cannot be compared directly. As such, we also expressed neutralization capability of the antivenom in terms of P (potency), which is the mass of venom that is completely neutralized per unit volume of antivenom, as defined by Morais et al. [12]. Potency gives a better estimate of the relative efficacy of antivenoms than comparing ED_50_ values when different challenge doses were used in the determination of ED_50_, as P is theoretically independent of the amount of challenge doses. Nevertheless, since the relationship between antivenom neutralizing capability versus venom challenge dose is not necessarily linear due to the complexity of venom and antibodies composition, comparing the P values of antivenom when using different challenge doses does have its limitation.

### Comparison of monovalent antivenoms versus polyvalent antivenoms

The relative merit of monovalent antivenoms versus polyvalent antivenoms has been the subject of much discussion [14] and there are some authors who suggested that monovalent antivenoms are generally more potent than polyvalent antivenoms and less likely to cause adverse reactions as it may involve administration of a lower quantity of antivenom IgG that with a polyvalent antivenom. Several studies have shown, however, that this is not necessarily true [14], [15]. Our results here demonstrated that the quality and neutralization capabilities of polyvalent antivenom are not necessary inferior to that of monovalent antivenoms. Here we compared the protein composition of the *N. kaouthia* monovalent antivenom(NKMAV) and Neuro Polyvalent Snake Antivenom (NPAV), as well as the *in vitro* neutralization potency of the two antivenoms against venoms from five common Asiatic cobras and *O. hannah* (Table 2). The results show that both antivenoms are devoid of high molecular weight aggregates, the compounds that are usually associated with adverse reactions. The protein contents of the two antivenoms are both relatively low (20.3 mg/mL and 12.5 mg/mL respectively, for NPAV and NKMAV). The neutralization potencies of NKMAV and NPAV against *N. kaouthia* (Thailand) venom (the venom used to raise both antivenoms) are essentially the same. This, however, is not surprising since according to the manufacturer, monovalent antivenoms (including NKMAV) were later added to the purified polyvalent antivenom to ensure the neutralizing potency of the NPAV was comparable with the neutralizing potencies of the respective monovalent antivenoms. What is interesting is that, the neutralization potencies of both antivenoms against four Asiatic cobra venoms tested are comparable. NPAV, however, is much more potent than NKMAV in neutralizing *O. hannah* venom. This is to be expected as *O. hannah* venom was included in the immunogen mixture used in raising NPAV. These observations are in accordance with the conclusion drawn by Raweerith and Ratanabanangkoon [15].

### Neutralization potency of NPAV against venoms of Malaysian elapids

NPAV was raised using the four common elapid venoms in Thailand: *N. kaouthia* (Thailand), *O. hannah*, *B. candidus* and *B. fasciatus*. Our results showed that the polyvalent antivenom could effectively neutralize venoms of the same four species that originated from Malaysia. It is interesting that the polyvalent antivenom could effectively neutralize venom of *N. kaouthia* from Malaysia too. According to Wüster and Thorpe [16], the composition of the venom of Thai *N. kaouthia* is substantially different from that of the Malaysian *N. kaouthia*, the former appears to be more neurotoxic, while the latter more necrotic. It was suggested that this difference in the toxin composition may result in antivenom incompatibility. Our results, however, showed that while the two *N. kaouthia* venoms did differ substantially in their venom *i.v.* LD_50_, the polyvalent antivenom that was raised from Thai *N. kaouthia* venom (together with other Thai elapid venoms) could effectively neutralize the venom of Malaysian *N. kaouthia*, albeit with a moderately lower ED_50_. The same cross-neutralization potency was also observed with the monovalent *N. kaouthia* antivenom.

In addition to the four elapid venoms mentioned, NPAV also effectively neutralized the venoms of the other Malaysian kraits *B. flaviceps* and *B. fasciatus*, as well as the medically important Equatorial spitting cobra *N. sumatrana*. Thus, the results of our *in vitro* neutralization studies suggest that NPAV, which is prepared from Thai elapid venoms, can be useful in the treatment of elapid envenoming in Malaysia, since the polyvalent antivenom can effectively neutralize venoms of all medically important elapids in Malaysia.

### Neutralization potencies of NPAV against other Asiatic cobra venoms

Our results showed that NPAV was effective (P>0.5 mg/mL) against other Asiatic cobra venoms, including the venoms of the three Southeast Asian spitting cobras *N. siamensis*, *N. sumatrana* and *N. sputatrix*, as well as the venoms of the Central Asia cobra *N. oxiana* and Chinese cobra, *N. atra*. Earlier report claimed that Chinese cobra *N. atra* venom was poorly neutralized by other commercial cobra antivenoms [17]. NPAV, however, was only weakly effective against the venom of *N. philippinensis*, the highly neurotoxic (lethal) Philippine cobra. The low neutralization potency of 0.11 mg/mL would mean that more than 50 vials of the antivenom may be required for the victim, as the amount of venom injected during a cobra bite can be more than 50 mg (dry weight). Indian polyvalent antivenom (raised from *N. naja* venom) has also been reported to show poor neutralizing ability against *N. philippinensis* venom.

NPAV was only moderately effective against *N. naja* venoms from the Indian subcontinent with the exception of one Sri Lankan *N. naja* venom sample (mixtures collected from several adult *N. naja*). The variation suggests geographical differences in *N. naja* venom.

Thus, our results suggest that NPAV is effective (P>0.5 mg/mL) in neutralizing the venoms of Asiatic cobras from Southeast Asia (except the Philippines cobra), Central Asia and China, but only moderately effective against venoms from the cobras from the Indian subcontinent. These observations can be used as the basis for the design of a polyvalent antivenom with a broader spectrum of cross-neutralization. For example, if venoms of *N. philippinensis*, *N. naja* and *B caeruleus* were added to the immunogen mixture to raise NPAV, the resulting polyvalent antivenom may well be an effective Pan-Asian polyvalent cobra and krait antivenoms.

### Neutralization potencies of NPAV against African cobra venoms

Five common African cobra venoms, including venoms from three spitting cobras (*N. nigricollis*, *N. nubiae* and *N. katiensis*) were selected for this study. NPAV could neutralize effectively the venoms from *N. melanoleuca*, but is moderately effective against that of the spitting cobras *N. nigricollis* and *N. nubiae*, and weakly or not effective against venom of the highly lethal *N. haje* and the spitting cobra *N. katiensis*. We have not carried out a thorough study of the neutralization potency of NPAV against African cobra venoms, as many of the venoms are not available to us. Nevertheless, the results showed that there are still substantial cross-neutralizations between major venom toxins of the Asiatic and the African *Naja*, despite the fact that the Asian group lies in a more distant branch from the African group in the phylogenetic dendrogram [18].

### Neutralization potency of NPAV against krait venoms

It is interesting to note that the NPAV could effectively neutralize the venoms of the three Southeast Asian kraits (*B. fasciatus*, *B. candidus* and *B. flaviceps*), but only moderately or weakly against the venoms of the other two kraits (*B. multicinctus* and *B. caeruleus*). The results indicate that krait venoms share enough common antigens among them to enable the NPAV raised against *B. fasciatus* and *B. candidus* to neutralize all 5 krait venoms tested. However, the low to moderate potency of NPAV against *B. multicinctus* and *B. caeruleus* venoms indicates significant differences in antigenicity of some of the venom toxins.

### *In vivo* neutralization potency of NPAV versus *in vitro* neutralization potency

For all four elapid venoms tested, the *i.m* LD_50_ values are comparable to the *i.v.* LD_50_, indicating that the main venom toxins could diffuse effectively from muscle to circulation, presumably because these toxins are mainly low molecular weight proteins (phospholipases A_2_ and the three-finger toxins). Also, the neutralization capability (as measured by ED_50_) of NPAV in the *in vivo* assay is comparable to that of *in vitro* assay, suggesting that neutralization potential of antivenom against elapid snakes measured by the usual *in vitro* neutralization assay does provide a good indication of its effectiveness in the *in vivo* situation.

### The protective effects of NPAV against cardiovascular depressant and neuromuscular blocking effects of *N. sputatrix* venom in anesthetised rats

To further examine the *in vivo* neutralization capability of the NPAV, we examined its ability to protect against *N. sputatrix* cobra venom-induced cardio-respiratory depressant and neuromuscular blocking effects in anesthetised rats. The antivenom (3 mL in total) was administered in 3 separate injections to minimize disturbing the cardio-respiratory parameters. It is well established that the major lethal toxins of *N. sputatrix* venom consist of polypeptide neurotoxins, polypeptide cardiotoxins and phospholipases A_2_ [19]. The *v*enom-induced cardio-respiratory depressant effect was likely due to the combined actions of polypeptide cardiotoxins and phospholipases A_2_ whereas the neuromuscular blocking effect was likely due mainly to the action of the venom polypeptide neurotoxins, although venom phospholipases A_2_ may also play a role. Thus, the ability of NPAV to effectively reverse the *N. sputatrix* venom-induced cardio-respiratory depressant and neuromuscular blocking effects in the rats demonstrated that the antivenom did contain specific antibodies that could effectively neutralize the major lethal toxins of *N. sputatrix* venom.

### Conclusions

In conclusion, the *in vitro* and *in vivo* neutralization studies indicated that Neuro Polyvalent Snake Antivenom (NPAV) effectively neutralized venoms from many Southeast Asian *Naja*, *Bungarus* and *Ophiophagus hannah* but less effective against the venoms of *Naja* from the India subcontinent and Africa, as well as the Asiatic *N. philippinensis*. This cross-neutralization information can be used as the basis for the design of broader-spectrum polyvalent cobra antivenom. The abilities of NPAV to protect against *N. sputatrix* venom-induced cardio-respiratory depressant and neuromuscular blocking effects confirmed that the antivenom effectively neutralized the major lethal toxins of *Naja* venoms. The antivenom may find potential application in the treatment of elapid bites in Southeast Asia, especially Malaysia, a neighboring nation of Thailand. Nevertheless, the applicability of NPAV in the treatment of cobra and krait envenomations in Southeast Asia needs to be confirmed by clinical trials, as it is known that antivenom that has been proved effective in murine model is not necessarily effective in treating human victims [20].
